# CRISPR/Cas9-mediated knockout of *c-REL* in HeLa cells results in profound defects of the cell cycle

**DOI:** 10.1371/journal.pone.0182373

**Published:** 2017-08-02

**Authors:** Carsten Slotta, Thomas Schlüter, Lucia M. Ruiz-Perera, Hussamadin M. Kadhim, Tobias Tertel, Elena Henkel, Wolfgang Hübner, Johannes F. W. Greiner, Thomas Huser, Barbara Kaltschmidt, Christian Kaltschmidt

**Affiliations:** 1 Department of Cell Biology, University of Bielefeld, Bielefeld, Germany; 2 AG Molecular Neurobiology, University of Bielefeld, Bielefeld, Germany; 3 Biomolecular Photonics, University of Bielefeld, Bielefeld, Germany; Universita degli Studi di Napoli Federico II, ITALY

## Abstract

Cervical cancer is the fourth common cancer in women resulting worldwide in 266,000 deaths per year. Belonging to the carcinomas, new insights into cervical cancer biology may also have great implications for finding new treatment strategies for other kinds of epithelial cancers. Although the transcription factor NF-κB is known as a key player in tumor formation, the relevance of its particular subunits is still underestimated. Here, we applied CRISPR/Cas9n-mediated genome editing to successfully knockout the NF-κB subunit *c-REL* in HeLa Kyoto cells as a model system for cervical cancers. We successfully generated a homozygous deletion in the *c-REL* gene, which we validated using sequencing, qPCR, immunocytochemistry, western blot analysis, EMSA and analysis of off-target effects. On the functional level, we observed the deletion of *c-REL* to result in a significantly decreased cell proliferation in comparison to wildtype (wt) without affecting apoptosis. The impaired proliferative behavior of *c-REL*^-/-^ cells was accompanied by a strongly decreased amount of the H2B protein as well as a significant delay in the prometaphase of mitosis compared to *c-REL*^+/+^ HeLa Kyoto cells. *c-REL*^-/-^ cells further showed significantly decreased expression levels of *c-REL* target genes in comparison to wt. In accordance to our proliferation data, we observed the *c-REL* knockout to result in a significantly increased resistance against the chemotherapeutic agents 5-Fluoro-2’-deoxyuridine (5-FUDR) and cisplatin. In summary, our findings emphasize the importance of c-REL signaling in a cellular model of cervical cancer with direct clinical implications for the development of new treatment strategies.

## Introduction

Cervical cancer is an epithelial cancer, also called carcinoma, and the fourth common cancer in women worldwide with an estimated 5-year survival rate of 70% following diagnosis [1, 2]. Based on the degenerated cell type in the uterus, cervical cancer can be classified into squamous cell cancer and adenocarcinoma [1]. The most common reason for cervical cancer is an infection by the human papilloma virus (HPV), namely by HPV 16 and HPV 18 causing malignant transformations or carcinogenesis in 85% of the diagnosed cases [3, 4]. Treatment strategies of cervical cancer highly depend on the stage of progression and range from radiotherapy and surgery [5] to chemotherapy with cisplatin or 5-fluorouracil (5-FU) [6, 7].

Discovered in 1986 [8, 9], the transcription factor nuclear factor kappa-light-chain-enhancer of activated B-cells (NF-κB) has been shown to play a key role in various cellular processes as cell growth, differentiation, apoptosis, inflammation, learning and memory as well as immunity [10, 11]. Given the importance of NF-κB in these processes, deregulation of its signaling is directly associated to the formation of tumors and cancer progression [12–14], particularly regarding breast cancer [15] and cervical carcinomas [1]. In 2003, Nair and coworkers showed a constitutive activation of the NF-κB subunit p65 during human cervical cancer progression. Here, NF-κB p65 was demonstrated to be particularly activated in high-grade squamous intraepithelial lesions and squamous cell carcinomas of the human uterine cervix [16]. Next to NF-κB p65, the subunit c-REL was shown to possess a key role in tumor formation. Initial studies demonstrated severe B-cell lymphomas in chickens infected with avian reticuloendotheliosis comprising V-REL [17]. Expression of wildtype human *c-REL* in primary chicken spleen cell cultures was likewise shown to result in malignant transformation events [18], although respective mutations increasing the oncogenicity of the c-REL protein in the avian system were not observable in human cancers (reviewed in [19]). However, amplification of *c-REL* was observed in a broad range of human B-cell lymphomas [20, 21]. In terms of human cervical cancer, Shehata and coworkers demonstrated a 6-fold slowed cell growth in cultivated cervical cancer cells by expression of the *c-REL* homolog Xrel3 from *Xenopus laevi* [22]. Accordingly, downregulation of *c-REL* by small interfering RNA was shown to result in reduced proliferation of human keratinocytes [23], directly correlating *c-REL* signaling to impaired cell cycle progression in a non-cancerous environment. Expression of the *c-REL* homolog Xrel3 in human cervical cancer cells was further shown to lead to anti- or pro-apoptotic effects during cisplatin-treatment in a concentration-dependent manner. These findings emphasize the importance of *c-REL*-signaling in resistance of cervical carcinoma to chemotherapeutic agents [24].

The present study further extends these promising findings by showing a profound overexpression of *c-REL* in cancers located in human ovary, cervix and endometrium using database mining. To investigate the role of *c-REL* in human cervical cancers in more detail, we applied CRISPR/Cas9n-mediated genome editing in a multiplex way to delete *c-REL* in HeLa Kyoto cells. Initially discovered as a part of adaptive immune system of bacteria and archaea [25], the clustered regularly interspaced short palindromic repeats (CRISPR) system has been developed to a state-of-the-art technique for editing the human genome [26, 27]. Applications of the CRISPR/Cas9-system particularly include cancer modeling [28] or knockout studies using human cancer cell lines [27, 29]. In the present study, we applied a Cas9 nickase mutant (Cas9n) inducing single-strand breaks to minimize the possibility of off-target cleavage in turn increasing the specificity of genome editing [30].

Using the CRISPR/Cas9n approach, we successfully deleted the *c-REL* gene on chromosomes 2 of HeLa Kyoto cells (*c-REL*^-/-^). In comparison to wildtype, *c-REL*^-/-^ HeLa Kyoto cells showed a significantly decreased proliferation accompanied by strongly reduced amounts of histone H2B, a delay in the prometaphase of mitosis and decreased expression levels of *c-REL* target genes. We further observed a significantly increased resistance against the chemotherapeutic agents 5-Fluoro-2’-deoxyuridine (5-FUDR) and cisplatin in HeLa Kyoto cells with *c-REL* deletion compared to wildtype (wt). Our findings emphasize the importance of *c-REL* signaling in a cellular model of cervical cancer with direct clinical implications concerning the resistance of cervical carcinoma to chemotherapeutic agents.

## Materials and methods

### Target design and cloning

The design of the sgRNAs was done using the CRISPR/Cas9n Target Online Predictor from University of Heidelberg (crispr.cos.uni-heidelberg.de). The gene sequence was taken from Ensembl Genome Browser (ensembl.org). Two nicking pairs were designed resulting in two double strand breaks creating a deletion. Nicking pairs were chosen according to the criteria described by Ran and coworkers [30]. All oligos designed were cloned into one vector essentially as described [31].

### Cell culture and transfection

HeLa Kyoto cells [32] were cultured in Dulbecco's Modified Eagle's Medium (DMEM) (Sigma Aldrich, Taufkirchen, Germany) containing high glucose (25 mM), and sodium pyruvate (1 mM). This medium was supplemented with 10% (v/v) heat-inactivated fetal calf serum (FCS) (VWR, Darmstadt, Germany), 2 mM L-glutamine (Sigma Aldrich), 100 U/ml Penicillin/Streptomycin (P/S) (Sigma Aldrich), and 0.5 mg/ml geneticin (G418) (Sigma Aldrich). Cells were cultivated at 37°C with 5% CO_2_ at saturated humidity.

Transfection of HeLa Kyoto cells (3 x 10^5^ cells / transfection) was performed by electroporation using Amaxa Cell Line Nucleofector Kit R (Lonza, Basel, Schweiz) according to the manufacturer’s protocol. 48 hours after transfection knockout generation was checked by genomic PCR and cells were used for limiting dilution to obtain clonal *c-REL* knockout cells.

### Genomic PCR and Native PAGE

For cell lysis, cells were harvested at 300 g for 5 min and resuspended in cell lysis buffer (0.1 μg/mL gelatine, 50 mM KCl, 1.5 mM MgCl_2_, 0.45% NP40, 10 mM TRIS pH 8.3, 0.45% TWEEN 20). Proteinase K (20 mg/ml, Serva Electrophoresis, Heidelberg, Germany) was added followed by incubation of the cell lysate for at least 1 h at 55°C and 5 min at 95°C. 2 μL were used for PCR (*c-REL* primers: Fw 5´-TGCATTTTCATTTTCAGTGAATGGT-3´, Rev 5´-ACCTGTGGAGATGACTGTGAAG-3´). Resulting bands on agarose gels were extracted using NucleoSpin Gel and PCR Clean up Kit (Macherey Nagel) according to manufacturer’s guidelines and subsequently analyzed by sequencing.

For Native PAGE, DNA of the PCR product was denaturized and re-annealed (5 min at 95°C, -2°C/s from 95°C to 85°C and 0.1°C/s from 85°C to 25°C). PCR product was separated on a 10% native Polyacrylamide-gel for 2 h at 150 V. Gene Ruler DNA Ladder Mix (Thermo Fisher Scientific, Waltham, MA, USA) served as marker, gel was immersed in 0.05% ethidium bromide (Carl Roth GmbH, Karlsruhe, Germany) for 5 min prior to visualization.

### Quantitative real-time PCR

RNA isolation was done with NucleoSpin^®^RNA Kit (Macherey-Nagel) according to manufacturer’s guidelines. 500 ng RNA were used for cDNA synthesis. Quantitative real-time PCR (qPCR) was performed using SYBR Green Master Mix (Thermo Fisher Scientific). cDNA was diluted 1:50 and 2 μL/reaction were used as template. Primer sequences were 5´-CTCCTGACTGACTGACTGCG-3´ (Fw *c-REL* target deletion), 5´-TACGGGTTATACGCACCGGA-3´ (Rev *c-REL* target deletion), 5´-CCTGGAGCAGGCTATCAGTC-3´ (Fw *RELA*), 5´-CACTGTCACCTGGAAGCAGA-3´ (Rev *RELA*), 5´-ACATCAAGGAGAACGGCTTCG-3´ (Fw *RELB*), 5´-GACACTAGTCGGCCCAGG-3´ (Rev *RELB*), 5´-GCACCCTGACCTTGCCTATT-3´ (Fw *NFKB1*), 5´-GCTCTTTTTCCCGATCTCCCA-3´ (Rev NFKB1), 5´-CAACCCAGGTCTGGATGGTA-3´ (Fw *NFKB2*), 5´-CTGCTTAGGCTGTTCCACGA -3´ (Rev NFKB2), 5´-TGACAGTGAGCCCTGAAAGC-3´ (Fw *IKBKE*), 5´-CCGGATTTCCCACACTCTGA-3´ (Rev *IKBKE*), 5´-CGGAGACCCGGCTGGTATAA-3´ (Fw *TBK1*), 5´-ATCCACTGGACGAAGGAAGC-3´ (Rev *TBK1*), 5´-CTGAAAACGAACGGTGACGG-3´ (Fw *A20*), 5´-TCCAGTTGCCAGCGGAATTT-3´ (Rev *A20*), 5´-CAGGATAACGGAGGCTGGGATG-3´ (Fw *BCL2*), 5´-TTCACTTGTGGCCCAGATAGG -3´ (Rev *BCL2*), 5´-GCTTGGATGGCCACTTACCT-3´ (Fw *BCL-XL*), 5´-ACAAAAGTATCCCAGCCGCC-3´ (Rev *BCL-XL*), 5´-GCAAGTGGACATCAACGGGT-3´ (Fw *TGFB1*), 5´- TCCGTGGAGCTGAAGCAATA-3´ (Rev *TGFB1*), 5´-GTAGTGGAAAACCAGCAGCC-3´ (Fw *MYC*), 5´-AGAAATACGGCTGCACCGAG-3´ (Rev *MYC*), 5´-ATGGCAACGACTCCTTCTCG-3´ (Fw *ICAM-1*), 5´-GCCGGAAAGCTGTAGATGGT-3´ (Rev *ICAM-1*). Ct values were normalized to reference genes *GAPDH* (Fw 5´-CATGAGAAGTATGACAACAGCCT-3´, Rev 5´-AGTCCTTCCACGATACCAAAGT-3´), *RPLP0* (Fw 5´-TGGGCAAGAACACCATGATG-3´, Rev 5´-AGTTTCTCCAGAGCTGGGTTGT-3´) and *eEF2* (Fw 5´-AGGTCGGTTCTACGCCTTTG-3´, 5´-TTCCCACAAGGCACATCCTC-3´).

### Western blotting

For analysis of RELA and A20, *c-REL*^-/-^ and *c-REL*^+/+^ cells were treated with human recombinant TNFα (10ng/ml, Calbiochem, Merck, Darmstadt, Germany) for 24h prior to protein isolation. Protein extracts were made using cell lysis buffer (0.01 M TRIS, 3 mM EDTA, 1% SDS) and equal amounts of protein were separated by SDS-PAGE and transferred to a PVDF membrane. Membranes were blocked using PBS containing 0.05% Tween 20 and 5% milk powder and probed with primary antibodies (rabbit anti-c-REL (#4727), Cell Signaling Technology, Danvers, MA, USA); rabbit anti-p65 (#8242), Cell Signaling; mouse anti-A20 (sc-166692), Santa Cruz Biotechnology, Heidelberg, Germany) overnight at 4°C. Horseradish peroxidase-conjugated secondary antibodies were applied for 1h at room temperature and blots were subsequently developed using enhanced chemiluminescence.

### Electrophoretic mobility shift assay

Electrophoretic Mobility Shift Assay was performed using DIG Gel Shift Kit, 2nd generation (Deutschland Holding GmbH, Grenzach-Wyhlen, Germany) according to manufacturer’s guidelines. For *c-REL* probe sequence (5´-TCGAGGGCTCGGGCTTTCCATCTCTCGA-3´), *c-REL* binding site CGGGCTTTCC was assessed using the JASPAR Tool (jaspar.genereg.net). Protein isolation procedure and unspecific competitor sequence were applied as described by Tokunaga and coworkers [33]. PAGE was performed as described above.

### Immunocytochemistry and fluorescence imaging of H2B-mcherry

For immunostaining and imaging of H2B-mCherry cells were seeded and cultivated on coverslips. Fixation was done by adding 4% paraformaldehyde (PFA) for 10 min. After repetitive washing using phosphate-buffered saline (PBS), cells were either directly mounted with Mowiol/DABCO or carried over to immunostaining. For immunocytochemistry, cells were blocked and permeabilized using 0.02% PBST (PBS with Triton X-100) containing 5% goat serum for 30 min at RT. Primary antibody (rabbit anti-c-REL (#4727), Cell Signaling; mouse anti-CD54/ICAM MAB1379, Chemicon, Merck) was applied for 1 h at RT. After washing, cells were incubated with secondary antibody (goat anti-rabbit Alexa Fluor 647, Thermo Fisher Scientific) for 1 h at RT under exclusion of light. Finally, coverslips were mounted with Mowiol/DABCO. Imaging was done by confocal laser scanning microscopy (LSM 780, Carl Zeiss, Oberkochen, Germany) and image processing was done using Fiji) and Adobe Photoshop CS6 (Adobe Systems, San José, USA) or Corel Draw (Corel Corporation, Ottawa, Canada).

### Proliferation & survival assay

Proliferation was analyzed with Orangu Cell Proliferation Assay Kit (Cell Guidance Systems, Cambridge, UK) used following the manufacturer’s protocol. Cells were counted with Cellometer Auto T4 Cell Viability Counter (Nexcelom, Lawrence, USA). For a calibrating curve 1000, 2500, 5000, 7500, 10000 and 15000 wildtype cells were seeded and incubated for 24 h at 37°C. For correct cell number determination after one day, one well of each condition was recounted.

For survival assay 5000 cells in 100 μl were seeded one day before treatment. Cells were incubated with chemotherapeutic agents cisplatin (CDDP) (P4394, Sigma Aldrich) and 5-Fluoro-2´-deoxyuridine (5-FUDR) (Sigma Aldrich) for 21 h and subsequently Orangu Cell Proliferation Assay Kit was applied.

### Flow cytometric analysis of the cell cycle, apoptosis and histone H2B-mCherry

DNA content measurement for analyzing cell cycle parameters was performed according to Kaltschmidt and colleagues [34] by harvesting 1 x 10^6^ cells at 300 g for 5 min followed by fixation with 70% (v/v) ethanol. After centrifugation at 300 x g for 10 minutes, staining solution (PBS containing 1 mg/ml glucose (Carl Roth GmbH), 4´,6-diamidino-2-phenylindole (DAPI; 0.5 mg/ml; Sigma-Aldrich), and 100 Kunitz units RNaseA (Thermo Fisher Scientific) was applied for 60 min under exclusion of light.

For apoptosis measurement, 1 x 10^6^ cells were labeled with Annexin V-PE (Miltenyi Biotec, Bergisch Gladbach, Germany) according to the manufacturer’s instructions. For analysis of H2B-mCherry, 1 x 10^6^ *c-REL*^+/+^ and *c-REL*^-/-^ cells were harvested and directly applied for flow cytometric analysis without additional staining procedures.

DAPI or Annexin V-PE-labeled cells as well as unstained cells (H2B-mCherry) were analyzed using a Gallios^™^ 10/3 flow cytometer (Beckman Coulter, Brea, CA, USA). Data analysis was performed using FlowJo Software (TreeStar, Olten, Switzerland), doublet discrimination for cell cycle analysis was assured by appropriate gating strategies.

### Live cell imaging

We imaged H2B-mCherry alpha-tubulin-eGFP expressing HeLa Kyoto c-REL+/+ and c-REL -/- cells in growth conditions at 37 degrees for more than 20 hours with a DeltaVision Elite imaging system (GE Healthcare). At 20x magnification (Olympus UPlanSApo 20x 0.75), we recorded on a CoolSNAP HQ2 (Photometrics, USA) CCD camera 15 different lateral positions with 3 axial position with 1μm spacing for each c-REL+/+ and c-REL-/- cells respectively every 10 minutes for each fluorescent emission channels (LED excitation source 461-489nm, 553-597nm and emission filtered at 501-549nm, 603-647nm respectively). A DIC image was recorded for reference at each timepoint. The fluorescent images were deconvolved with the appropriate OTF in SoftWoRx (version 6.1.3, GE Healthcare), analysed with Fiji and figures were prepared with Omero.

### Promoter analysis

Sequence of promoter regions (1500 bp downstream and 100 bp upstream to respective ATG, 5000 bp downstream for c-Myc promoter) of interest were taken from Eukaryotic Promoter Database (epd.vital-ti.ch) for *Homo sapiens*. Binding sites for gene of interest in chosen promoter sequence were looked up using JASPAR Tool (jaspar.genereg.net). A relative score threshold of 85% was used. *RELA* and *c-REL* binding sites were compared in promoter regions of selected target genes.

### Statistics

All statistical tests were performed with PrismGraph Pad 5 (GraphPad Software, La Jolla, USA). Statistical significance of qPCR results and fluorescence intensity quantification was analyzed using unpaired t-test. Welch correction was performed, if variances were significantly different. Data of proliferation and survival assays were shown to be not normally distributed (Shapiro-Wilk test) and analyzed using Kruskal-Wallis test with Dunn post-hoc test.

## Results

### *c-REL* is overexpressed in human cervical cancers

**Fig 1:**
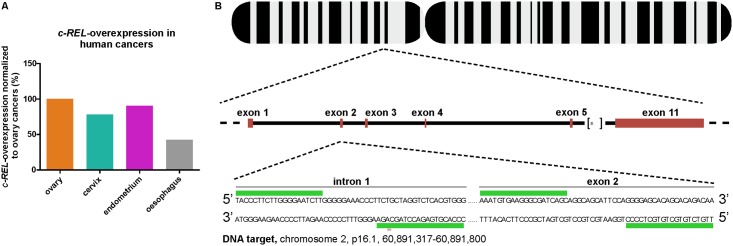
Assessment of *c-REL* overexpression in human cancers and target design of CRISPR/Cas9n-mediated *c-REL* knockout. **A**: Database mining revealed more profound overexpression of ***c-****REL* in cancers from human ovary, cervix and endometrium compared to oesophagus ([35], sancer.sanger.ac.uk; 02-14-2017 16:00; 02-21-2017 15:10). **B**: Target design showing the proposed ***c-****REL* knockout with an expected deletion around 450 bp targeting the intron 1-exon 2-boundary of the ***c-****REL* gene. The design was done with the CRISPR/Cas9n Target Online Predictor from the University of Heidelberg [36], crispr.cos.uni-heidelberg.de) and the gene sequence was taken from Ensembl Genome Browser (ensembl.org).

To assess the clinical implications of a *c-REL* knockout, we assessed levels of *c-REL* overexpression in human cancers by database mining using COSMIC [35]. We found *c-REL* to be profoundly overexpressed in human cancers, particularly within those located in human ovary, cervix and endometrium in comparison to oesophagus (Fig 1A, cancer.sanger.ac.uk; 02-14-2017 16:00; 02-21-2017 15:10). Due to their human cervix origin, we decided to apply HeLa Kyoto cells for the CRISPR/Cas9n-mediated *c-REL* knockout.

### Successful knockout of *c-REL* in HeLa Kyoto cells using CRISPR/Cas9n

**Fig 2:**
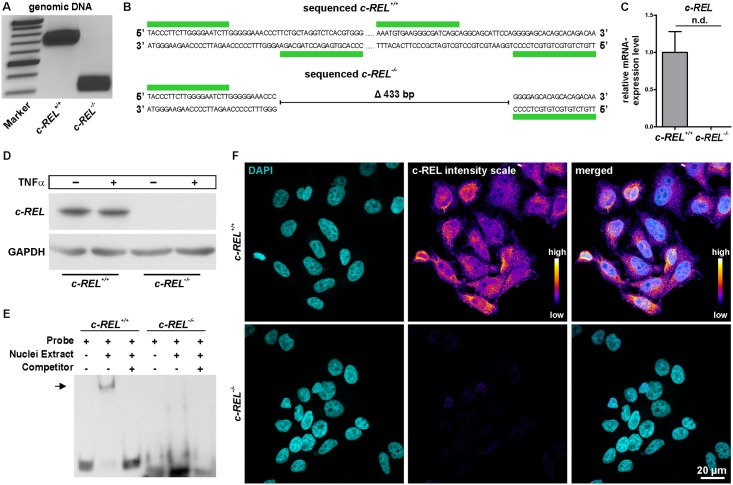
Successful validation of the *c-REL* knockout in HeLa Kyoto cells on DNA, mRNA and protein level. **A:** Genomic PCR depicting a profound deletion of the *c-REL* gene in the *c-REL* knockout clone (band at 300 bp) compared to the wt clone (band at 700 bp). **B:** Sequencing analysis confirmed the knockout in exon 2 of *c-REL*. **C:** qPCR with specific primers in targeted deletion of exon 2 showed no expression of *c-REL* on mRNA level in the *c-REL* knockout clone in comparison to wt. **D:** Western blot analysis validated the knockout of *c-REL* on protein level. **E:** Electrophoretic mobility shift assays (EMSA) showed DNA-binding of c-REL in HeLa Kyoto wt cells (arrow), which was not observable in the *c-REL* KO clone. **F:** Immunocytochemistry depicted a nearly complete loss of c-REL-protein in *c-REL* knockout clone compared to HeLa Kyoto wt cells.

To generate a *c-REL* knockout in HeLa Kyoto cells, we designed a target deletion around 450 bp between intron 1 and exon 2 of chromosome 2 using the CRISPR/Cas9 Target Online Predictor tool (Fig 1B, [36], crispr.cos.uni-heidelberg.de). All designed oligonucleotides were cloned into an all-in-one vector according to Golden Gate Assembly method (mCRISPR, [31]) allowing easier generation of knockouts. Genomic PCR depicted a profound deletion of the *c-REL* gene in clonally grown HeLa Kyoto cells after transfection with the constructed CRISPR/Cas9 vector in comparison to untransfected HeLa Kyoto wt cells (Fig 2A). Sequencing analysis confirmed the knockout of around 433 bp in exon 2 of *c-REL* within the transfected HeLa Kyoto clone.

### CRISPR/Cas9n-mediated *c-REL* knockout can be validated on mRNA and protein level

After initial analysis of the *c-REL* knockout on DNA level, we assessed the expression level of *c-REL* in the HeLa Kyoto knockout clone by qPCR with primers in the targeted deletion. In contrast to HeLa Kyoto wt cells showing a robust expression of *c-REL* on mRNA level, no expression was detectable in the *c-REL* knockout clone (Fig 2C). Notably, we analysed the top three predicted exonic off-targets and detected no significant signs of off-target effects in the *c-REL* kockout clone (S1 Fig).

In contrast to HeLa Kyoto wt cells, no c-REL protein was detectable in knockout cells by western blot analysis even after TNFα-dependent stimulation, confirming the knockout of *c-REL* on protein level (Fig 2D). Assessing a potential loss in functionality of the c-REL protein, we investigated DNA binding activity of c-REL using electrophoretic mobility shift assay (EMSA). *c-REL*^*-/-*^ cells showed no DNA-binding activity of c-REL (Fig 2E), whereas a clear shift was observable using HeLa Kyoto wt cells (Fig 2E, arrow). Immunocytochemistry further validated the *c-REL* knockout in the transfected HeLa Kyoto clone by showing a nearly complete loss of c-REL protein in comparison to HeLa Kyoto wt cells (Fig 2F).

### CRISPR/Cas9n-mediated deletion of *c-REL* results in a decreased proliferation of HeLa Kyoto cells without affecting apoptosis

**Fig 3:**
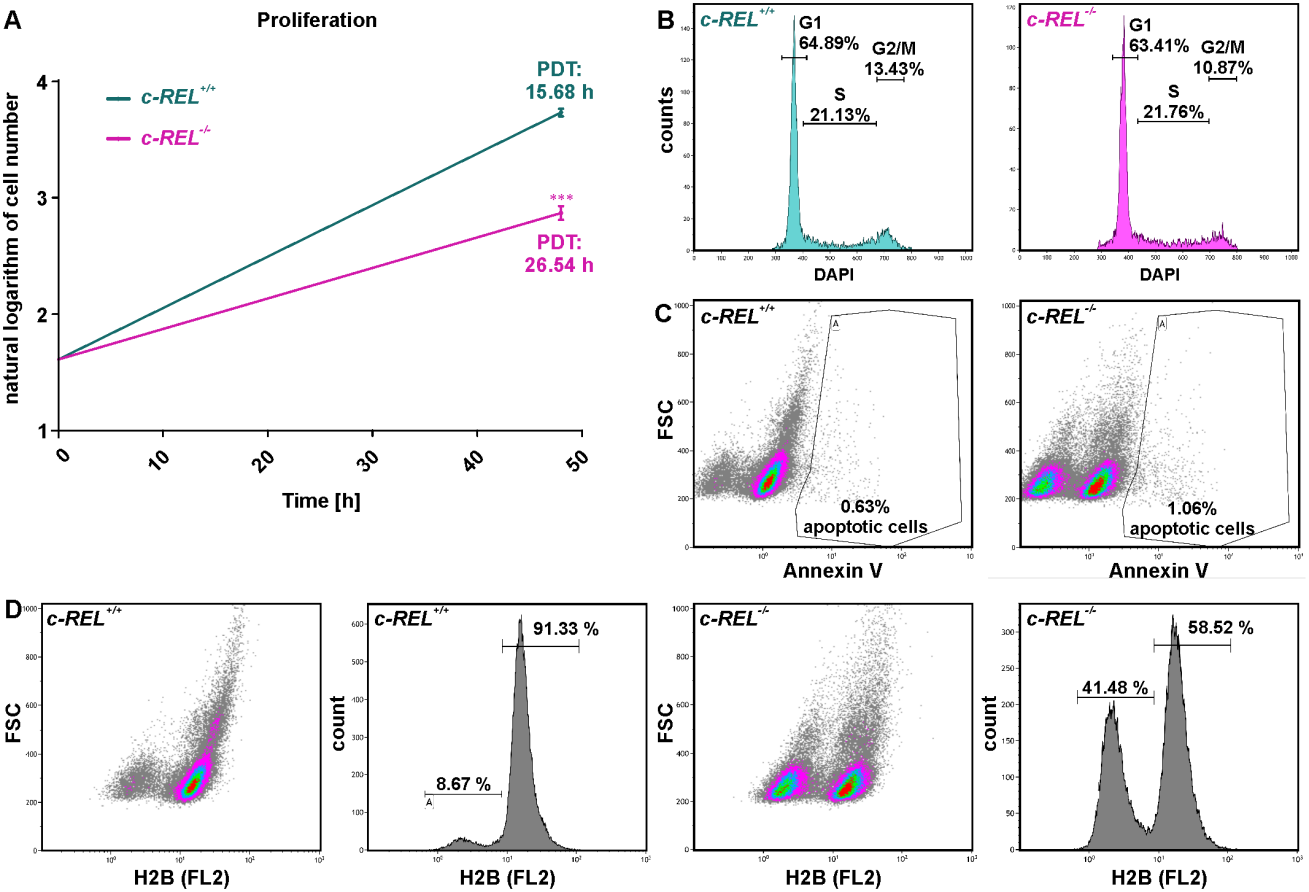
CRISPR/Cas9-mediated deletion of *c-REL* results in a decreased proliferation of HeLa Kyoto cell accompanied by strongly reduced amounts of histone H2B. **A:** Cell number assessed by Orangu Cell Proliferation Assay Kit (Cell Guidance Systems) set against cultivation time showed a strongly increased population doubling time of *c-REL* knockout cells compared to wt HeLa Kyoto cells. PDT: Population doubling time. **B:** Flow cytometric DNA content measurements of DAPI-stained *c-REL* knockout cells showed a decrease in the amount of mitotic cells in *c-REL* knockout cells compared to wildtype. **C:** Flow cytometric analysis of Annexin V-stained *c-REL*^-/-^ and wt HeLa Kyoto cells revealed only slightly increased amounts of apoptotic cells upon *c*-REL deletion in comparison to wt. **D:** Flow cytometric analysis of histone H2B-mCherry showed a strongly decreased amount of the H2B protein in 41.48% of *c-REL*^-/-^ HeLa Kyoto cells, which was observable in only 8.67% of HeLa Kyoto wt cells.

We next analyzed potential effects of the *c-REL* knockout on proliferation and apoptosis of HeLa Kyoto cells. Using Orangu Cell Proliferation Assay Kit (Cell Guidance Systems), proliferation of *c-REL* knockout and wt cells was assessed after 2 days. HeLa Kyoto *c-REL*^-/-^ cells showed a strongly increased population doubling time of 26.54 h compared to wt HeLa Kyoto cells displaying a population doubling time of 15.68 h (Fig 3A). This robustly decreased proliferative behavior of *c-REL* knockout cells was accompanied by a 0.81 fold decrease in the amount of mitotic cells compared to wildtype, as shown by cell cycle analysis using flow cytometric DNA content measurements (Fig 3B). However, we observed only slightly increased levels of Annexin V-positive apoptotic cells in *c-REL*^-/-^ cells compared to wt cells (Fig 3C), indicating the effect of the *c-REL* knockout on proliferation of HeLa Kyoto cells to be apoptosis-independent.

### *c-REL*^-/-^ HeLa Kyoto cells reveal strongly reduced levels of histone H2B accompanied by a significantly delayed prometaphase or complete arrest of the cell cycle

**Fig 4:**
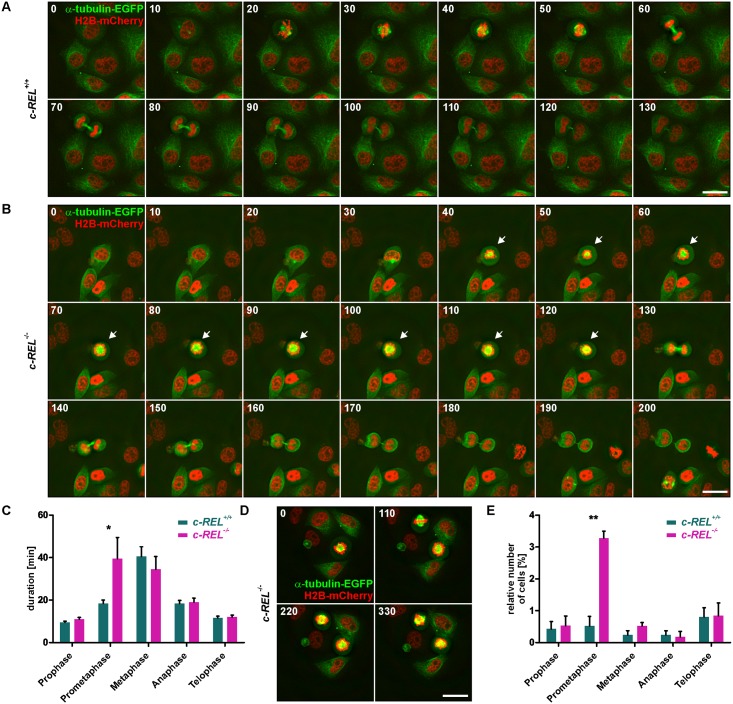
Knockout of *c-REL* leads to a significantly delayed prometaphase or even complete arrest of the cell cycle. **A-B:** Live cell imaging of *c-REL*^-/-^ and *c-REL*^+/+^ cells showed delayed duration of the prometaphase in *c-REL*^-/-^ (arrows) in comparison to wildtype. Mitosis was visualized by H2B-mCherry and alpha-tubulin-EGFP. **C:** Quantification of life cell imaging validated the significant delay of *c-REL*^-/-^ *in* length of the prometaphase (39.50 ± 9.96 min) in comparison to wt (18.42 ± 1.58 min) (n = 20). **D:** Exemplary images of *c-REL*^-/-^ cells arresting during mitosis without entry of the G2 phase of the cell cycle. **E:** Fluorescence imaging of H2B-mCherry in fixed cells displayed a significantly increased amount of *c-REL*^-/-^ HeLa Kyoto cells within the prometaphase compared to wt cells. (>1000 cells quantified per genotype, n = 3). Scale bar: 25 μm.

Assessing the reduced proliferative behavior of *c-REL*^-/-^ HeLa Kyoto cells in more detail, we analyzed the protein level of histone H2B, which is fused to mCherry in HeLa Kyoto cells [32]. Flow cytometric analysis of H2B-mCherry showed a strongly decreased amount of the H2B protein in 41.48% of *c-REL*^-/-^ HeLa Kyoto cells. On the contrary, we observed a reduced H2B protein level in only 8.67% of HeLa Kyoto wt cells (Fig 3D). Taking advantage of the H2B-mCherry and alpha-tubulin-EGFP fusion in HeLa Kyoto cells, we further visualized the different stages of mitosis in fixed cell samples and living cells. Fluorescence imaging of fixed cells revealed a significantly increased amount of *c-REL*^-/-^ HeLa Kyoto cells within the prometaphase compared to wt cells (Fig 4E). We investigated this effect of the *c-REL* deletion in more detail by live cell imaging. Here, *c-REL*^-/-^ cells showed a length of the prometaphase of 39.50 ± 9.96 min, which was significantly delayed in comparison to wt cells revealing a duration of the prometaphase of 18.42 ± 1.58 min (Fig 4A–4C, S1 Movie). In addition, we observed only 5.4% of wt cells but 25.7% of *c-REL*^-/-^ cells (n = 40) to arrest during mitosis without entry of the G2 phase of the cell cycle (Fig 4D, S2 Fig).

### *c-REL* knockout leads to significantly decreased expression levels of NF-κB family members and cell cycle-associated *c-REL* target genes

**Fig 5:**
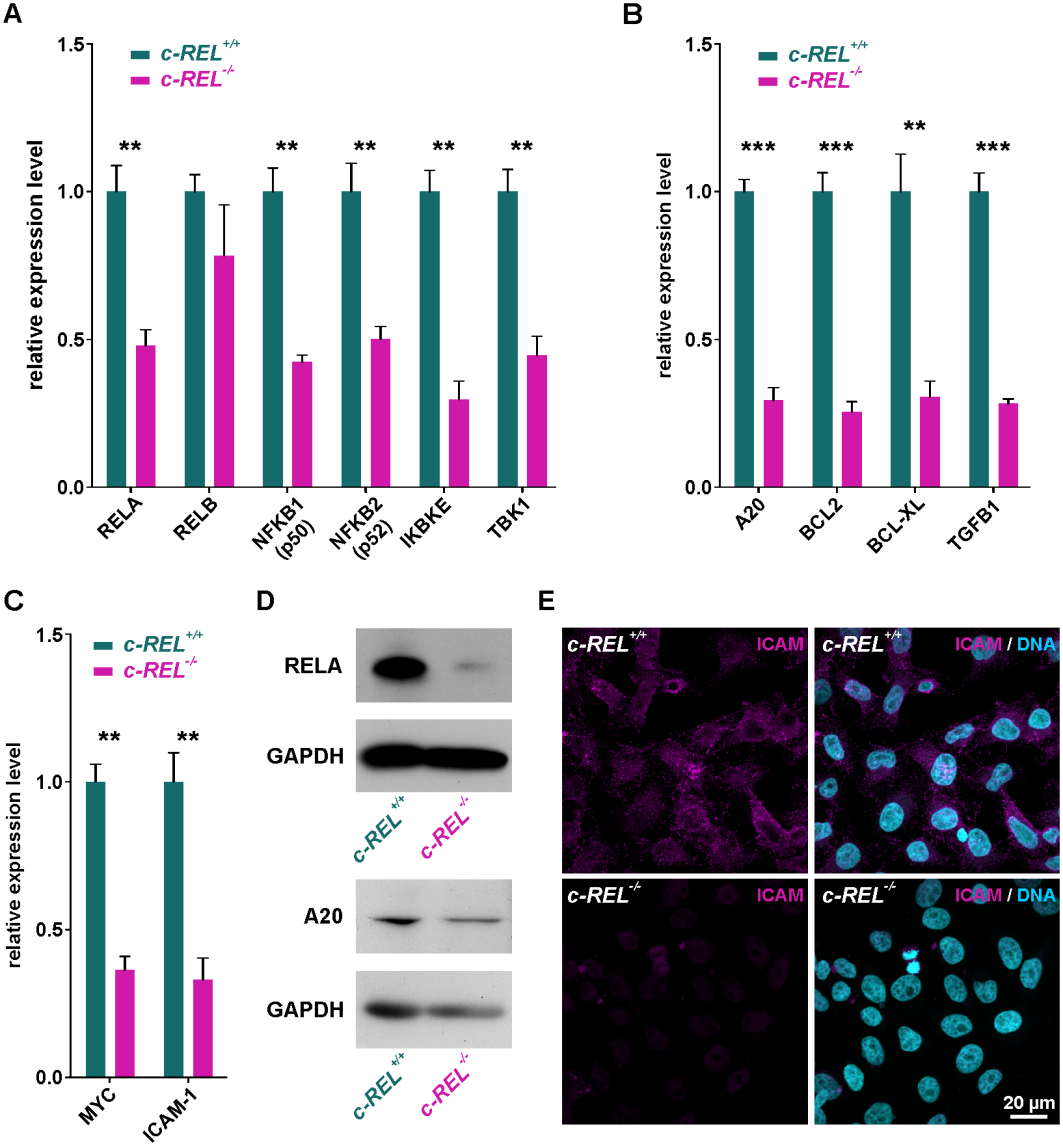
*c-REL* knockout leads to significantly decreased expression levels of NF-κB family member and cell cycle-associated genes. **A:** qPCR analysis showing significantly decreased mRNA levels of NF-κB family members *RELA*, *NFKB1 (p50)*, *NFKB2* (*p52)*, *IKBKE* and *TBK1* in *c-REL* knockout cells compared to wildtype cells. **B-C:** Expression levels of cell cycle-related *c-REL* target genes *A20*, *BCL2*, *BCL-XL* and *TGFB1* and *c-REL* target genes *MYC* and *ICAM-1* were significantly decreased in *c-REL* knockout cells in comparison to HeLa Kyoto wildtype cells. **D:** Western blot analysis validated the reduced expression levels of RELA and A20 in *c-REL*^-/-^ cells in comparison to wt on protein level. WB were performed after TNFα-dependent stimulation of *c-REL*^-/-^ and *c-REL*^+/+^ cells. **E:** Immunocytochemistry revealed a strongly decreased protein amount of ICAM in *c-REL*^-/-^ cells in comparison to wt.

Analyzing effects of the *c-REL* knockout in HeLa Kyoto cells on other NF-κB family members, we assessed respective gene expression levels by qPCR. *c-REL* knockout cells revealed significantly decreased mRNA levels of *RELA*, *NFKB1 (p50)*, *NFKB2* (*p52)*, IκB-Kinase ε (*IKBKE*) and TANK-binding kinase 1 (*TBK1*) compared to wildtype cells (Fig 5A). On the contrary, expression levels of *RELB* were not significantly affected in the *c-REL* knockout clone (Fig 5A).

In accordance to the observed decrease in proliferation and in *c-REL* knockout cells, we further observed significantly decreased mRNA levels in cell cycle-related *c-REL* target genes. In particular, expression levels of A20 (*TNFAIP3*), B-cell lymphoma 2 (*BCL2*), B-cell lymphoma-extra large (*BCLXL*, *BCL2L1*) and transforming growth factor beta 1 (*TGFB1*) were found to be significantly decreased in comparison to HeLa Kyoto wildtype cells (Fig 5B). In addition, expression levels of the *c-REL* target genes *MYC* and Intercellular Adhesion Molecule 1 (*ICAM-1*) were likewise significantly decreased compared to wildtype HeLa Kyoto cells (Fig 5C).

Promoter analysis was further performed using the JASPAR Tool (jaspar.genereg.net) to validate the analyzed genes to be direct *c-REL* target genes. Binding sites for *c-REL* and *RELA* were analyzed in each promoter region and their presence confirmed *IKBKE*, *TBK1*, *A20*, *BCL2*, *BCL-XL*, *TGFB1*, *MYC* and *ICAM-1* to be direct *c-REL* target genes (S3 Fig).

To validate the decreased expression levels of *c-REL* target genes in *c-REL*^-/-^ HeLa Kyoto cells on protein level, we performed western blot analysis and immunocytochemistry. Western blot analysis revealed reduced amounts of RELA and A20 protein in *c-REL*^-/-^ cells in comparison to wt (Fig 5D). We further observed a nearly complete loss of ICAM protein in *c-REL*^-/-^ cells by immunocytochemistry, while HeLa Kyoto wt cells showed an unchanged amount of ICAM protein (Fig 5E).

### HeLa Kyoto cells with *c-REL* deletion show a significantly increased resistance against chemotherapeutic agents

**Fig 6:**
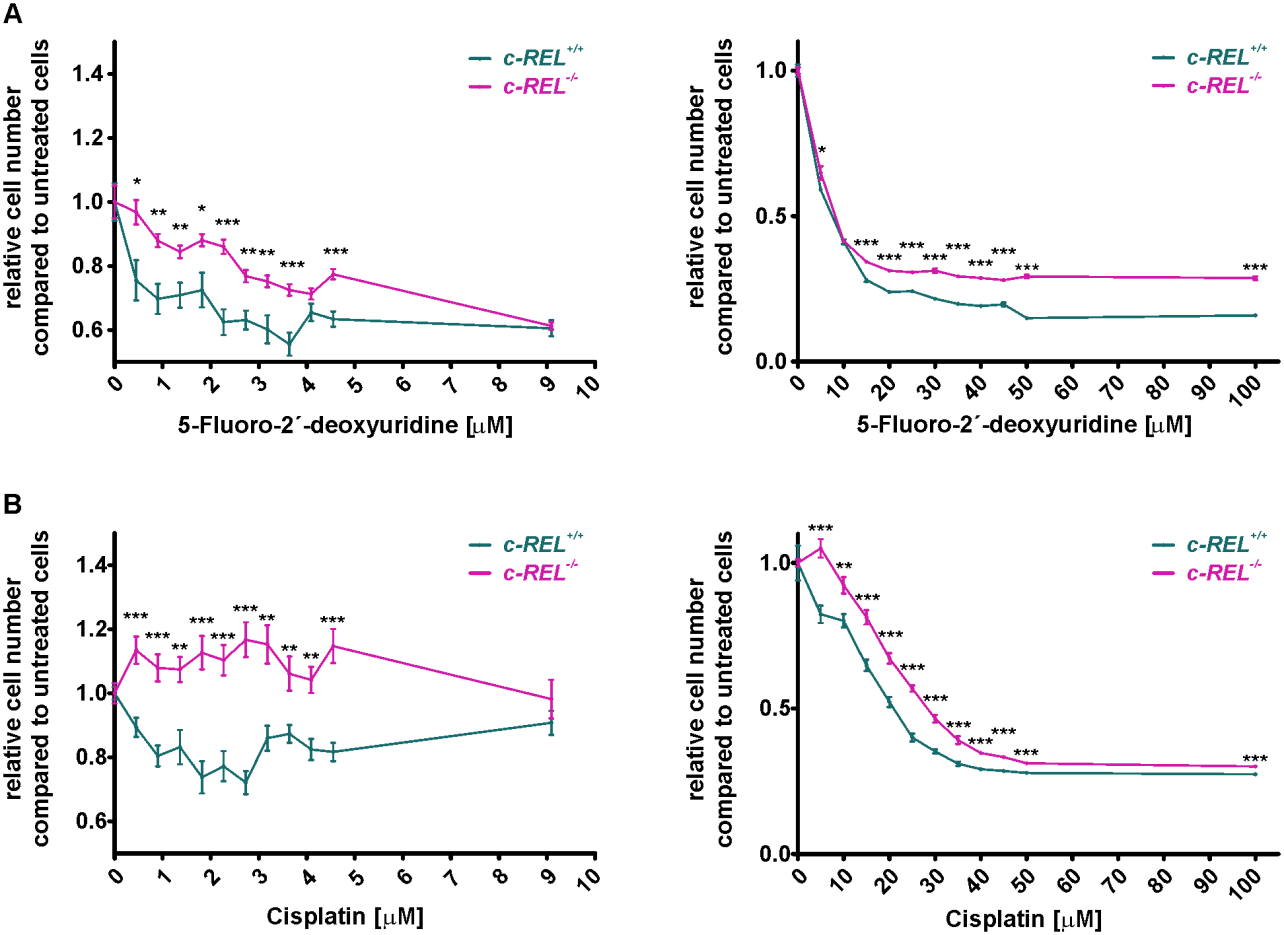
Significantly increased resistance against chemotherapeutic agents in HeLa Kyoto cells with *c-REL* deletion. **A:** Although both HeLa Kyoto wildtype and *c-REL* knockout cells showed cell death after treatment with 5-Fluoro-2’-deoxyuridine, the *c-REL* knockout clone showed significantly elevated cell numbers in comparison to wildtype cells. Cells were exposed to chemotherapeutic agents for 21 h, cell numbers were assessed using Orangu Cell Proliferation Assay Kit (Cell Guidance Systems) after 2 h of incubation. Cell number of untreated cells were set to 1 and used for comparison. **B:** Although cisplatin-treatment of 1–4 μM led to cell death of wildtype cells, survival of the *c-REL* knockout clone was significantly increased even in comparison to untreated control. Increasing concentrations of cisplatin (10–100 μM) affected survival of *c-REL* knockout cells, but cell numbers were still significantly elevated compared to wildtype. Cells were exposed to chemotherapeutic agents for 21 h, cell numbers were assessed using Orangu Cell Proliferation Assay Kit (Cell Guidance Systems) after 2 h of incubation. Cell number of untreated cells were set to 1 and used for comparison (n = 8).

With regards to the assessed overexpression of *c-REL* in human cancers (Fig 1A), potential clinical implications of the *c-REL* knockout were assessed by determining cell survival upon exposure to the chemotherapeutic agents 5-Fluoro-2’-deoxyuridine (5-FUDR) and cisplatin. Here, treatment with increasing concentration of 0.45–100 μM 5-FUDR for 21 h led to cell death of both *c-REL*^*-/-*^ and wildtype cells (Fig 6A).

However, 5-FUDR-treated *c-REL* knockout clone showed significantly increased cell numbers in comparison to wildtype cells, indicating a profoundly increased resistance against 5-FUDR (Fig 6A). We likewise observed this effect after treatment of HeLa Kyoto wildtype and knockout cells with cisplatin. While cisplatin-treatment of 0.45–4.5 μM led to cell death of wildtype cells, assessed cell numbers of the *c-REL* knockout clone were significantly increased even in comparison to untreated control (Fig 6B). Although exposure to increasing concentrations of cisplatin (10–100 μM) also resulted in cell death of *c-REL* knockout cells, cell number were still found to be significantly increased compared to wildtype, validating a robust resistance against cisplatin (Fig 6B).

## Discussion

The present study shows a successful CRISPR/Cas9n-mediated knockout of the NF-κB subunit *c-REL* in HeLa Kyoto cells. HeLa cells are one of the most frequently used model systems for epithelial and in particular cervical cancers [37–39]. Here, we observed a significantly decreased proliferation of *c-REL*^-/-^ cells accompanied by a significant decline in expression levels of NF-κB target genes in comparison to wt cells. HeLa Kyoto cells with *c-REL* deletion further revealed a significantly increased resistance against the chemotherapeutic agents 5-Fluoro-2’-deoxyuridine (5-FUDR) and cisplatin. These are commonly used as the trademarked chemotherapeutics Platinol^®^ and FUDR^®^ in the clinic.

With NF-κB being involved in many cellular processes [10, 11], a broad range of genes were described to be direct targets of NF-κB, including cytokines, chemokines, cell adhesion molecules, cell surface receptors, regulators of apoptosis and growth factors [40]. Interestingly, particular subunits of NF-κB were only rarely directly linked to specific target genes. In the present study, *c-REL*^-/-^ HeLa Kyoto cells showed significantly decreased expression levels of NF-κB family members *RELA*, *NFKB1*, *NFKB2* as well as *IKBKE* and *TBK1*. We also observed several *c-REL* binding sites in the promoter region of the *TBK1* and *IKBKE* gene, suggesting *TBK1* and *IKBKE* as direct *c-REL* target genes. In addition to their role in phosphorylating NF-κB p65 [41], TBK1 and IKBKE were in turn described to directly phosphorylate the C-terminal domain of the c-REL protein resulting in its nuclear accumulation [42]. Extending these promising findings, we suggest a positive feedback loop by *c-REL*-mediated expression of *TBK1* and IKBKE in turn leading to a pronounced activation of *c-REL*. Being also closely linked to the pathogenesis of breast cancer by promoting activation of NF-κB [43], targeting *IKBKE* may be an interesting future perspective for developing new treatment strategies against cervical cancer. Next to *TBK1* and *IKBKE*, *c-REL* knockout was observed to be sufficient to downregulate the expression of *MYC* in growing HeLa cells by more than 50% with the relative *MYC* expression being highly elevated in comparison to other target genes. Accordingly, Grumont and coworkers showed an impaired expression of *MYC* in stage III thymocytes with a *RELA*/*c-REL* double knockout [44]. We also demonstrated the presence of three *c-REL* binding sites in the downstream region of the *MYC* promoter, further validating *MYC* as a direct target gene of *c-REL*.

In comparison to wildtype, *c-REL*^-/-^ HeLa Kyoto cells further revealed a significantly decreased expression of *BCL-2*, *BCL-XL* and *A20*, which are commonly known as anti-apoptotic genes [45, 46]. In accordance to our promoter analysis depicting *c-REL*-binding sites, *BCL-XL* and *BCL2* were described to be direct *c-REL* target genes [47, 48]. Expression of *TGFB1*, already known as direct target of *c-REL* [49] and a common inducer of cell proliferation [50], was also significantly reduced in *c-REL*^-/-^ HeLa Kyoto cells. On functional level, we observed the *c-REL* knockout to result in a significantly reduced proliferation, which we suggest to be at least in part mediated by the depicted decline in pro-proliferative target gene expression. In consistence with these findings, knockdown of the *c-REL* target gene *IKBKE* in HeLa cells was also shown to result in a suppression of proliferation [51]. In human keratinocytes, small interfering RNA-mediated knockdown of c-REL was reported to directly affect cell cycle progression by cell cycle delay of the G2/M phase [23]. The present study further extended these findings by showing the CRISPR-Cas9n-mediated knockout of *c-REL* to result in a robustly delayed prometaphase of mitosis accompanied by strongly reduced levels of histone H2B protein. In addition, we observed a novel linkage between the decreased amount of histone H2B protein and the prolonged prometaphase in *c-REL*^-/-^ cells. In mice, silencing of c-Rel by siRNA was shown to lead to a reduction of mitosis in a B cell tumor cell line [52]. Grumont and coworkers likewise demonstrated a cell cycle arrest in B-cells of *c-Rel*^*-/-*^ mice [53]. Our present findings for the first time transfer these promising data to the human cancerous systems and provide deeper insights into the biology of cervical cancers in relation to *c-REL*-signaling. In this regard, we were also able to observe significantly reduced expression levels of *ICAM1* in *c-REL*^-/-^ HeLa Kyoto cells. Downregulation of this adhesion molecule was described to result in a suppression of human breast cancer cell invasion with the level of expression being directly correlated to their metastatic potential [54]. Accordingly, inhibition of MYC protein family members have been shown to induce regression of lung cancer in mice [55], suggesting the downregulation of *MYC* observed here likewise to be linked to the reduced proliferation of *c-REL*^-/-^ cells.

The NF-κB subunit *c-REL* is also directly linked to cancer development and progression. In 1999, Krappmann and colleagues described a constitutive NF-κB-activity with NF-κB-complexes containing RELA and c-REL in malignant cells derived from Hodgkin’s disease [56]. Whereas *c-REL* was currently discussed as being mutated in hematopoietic and lymphoid tumors [57], a high throughput database analysis performed in the present study including 3397 hematopoietic and lymphoid tumors detected mutations in only a few samples [35, 58]. In 2004, Futreal and coworkers described a ‘census’ of human cancers indicating mutations in >1% of genes of the human genome to contribute to cancer, although genes showing solely altered expression levels were not included in this initial ‘census’ [59]. Here, we applied database mining using the COSMIC database [35] and observed profound overexpression of *c-REL* in various human tumors (Table 1), which is in accordance to the observed amplification of *c-REL* in human B-cell lymphomas [20, 21]. Likewise in line with previous studies, *c-REL* can be considered as one of the most oncogenic members of the NF-κB family, in fowl reticuloendotheliosis virus also contains mutated oncogenic *v-Rel* [18, 19, 52, 60].

**Table 1:** Overexpression of *REL* in human cancers.

tissue type	% of *REL* overexpression	no. tested
**Ovary**	7.52	266
**Lung**	7.26	1019
**Urinary tract**	7.11	408
**Endometrium**	6.81	602
**Pancreas**	6.7	179
**Haematopoietic and lymphoid**	6.33	221
**Soft tissue**	6.08	263
**Cervix**	5.86	307
**Upper aerodigestive tract**	5.75	522
**Kidney**	5.5	600
**Thyroid**	5.46	513
**Large intestine**	4.92	610
**Stomach**	4.91	285
**Liver**	4.83	373
**CNS**	4.73	697
**Prostate**	4.62	498
**Breast**	3.71	1104
**Skin**	3.59	473
**Oesophagus**	3.2	125
**Adrenal gland**	2.53	79

In the present study, knockout of *c-REL* in a cellular model of cervical carcinoma resulted in a significantly increased resistance against the chemotherapeutic agents 5-FUDR and cisplatin. Due to direct interaction of cisplatin and 5-fluorouracil with the DNA, highly proliferating cells are exposed to DNA damage resulting in cell-cycle arrest and cell death [61, 62]. Thus, we suggest the reduced proliferation of HeLa *c-REL*^-/-^ cells to account for the observed increase in resistance against cisplatin and 5-FUDR. Although activation of NF-κB was also described to lead to a decreased sensitivity of cancer cells against chemotherapeutic treatment [63, 64], our present findings propose a subunit specificity of NF-κB in terms of chemoresistance. While a knockout of *c-REL* promoted survival of HeLa cells to chemotherapy, expression of the *c-REL* homolog *Xenopus* Xrel3 in cervical cancer cells treated with 5 μM cisplatin was shown to result in increased apoptosis [24].

In summary, our findings emphasize the importance of *c-REL*-signaling in a cellular model of cervical cancer particularly in terms of proliferation and resistance to chemotherapeutic agents. Considering the proposed NF-κB-subunit specificity of chemoresistance, we provide deeper insights into cervical cancer biology with direct clinical implications for the development of new treatment strategies.

## Supporting information

S1 Fig PAGE-analysis of top three predicted exonic off-targets revealed no signs of off-target effects in the *c-REL* knockout clone. (TIF)

S2 Fig Live cell imaging showing *c-REL*^-/-^ cells, which arrested during mitosis without entry of the G2 phase of the cell cycle. Scale bar: 25 μm.
(TIF)

S3 Fig Promoter analysis using the JASPAR Tool (jaspar.genereg.net) validated *IKBKE*, *TBK1*, *A20*, *BCL2*, *BCL-XL*, *TGFB1*, *MYC* and *ICAM*-1 to be direct *c-REL* target genes. *c-REL* binding site is shown in magenta, *RELA* binding site is depicted in cyan and common binding sites are shown in purple.
(TIF)

S1 Movie Live cell imaging of *c-REL*^-/-^ and *c-REL*^+/+^ cells showed delayed duration of the prometaphase in *c-REL*^-/-^ in comparison to wildtype. Mitosis was visualized by H2B-mCherry and alpha-tubulin-EGFP.
(MP4)
