# Supplementary figures and images for: CRISPR/Cas9-mediated knockout of c-REL in HeLa cells results in profound defects of the cell cycle
Source: PLoS One. 2017 Aug 2;12(8):e0182373. doi: 10.1371/journal.pone.0182373 (PMC5540532; doi:10.1371/journal.pone.0182373)

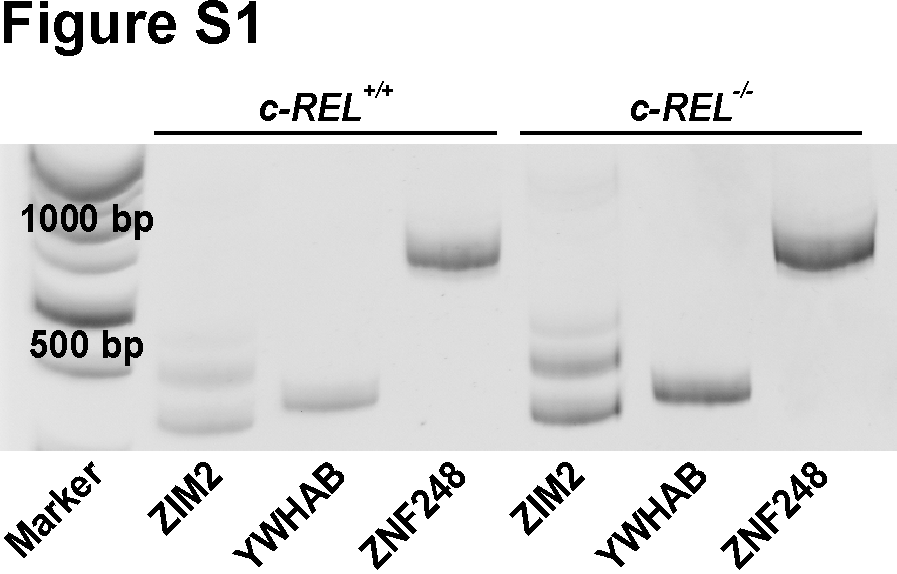

Supplement: S1 Fig — (TIF) [file pone.0182373.s001.tif]

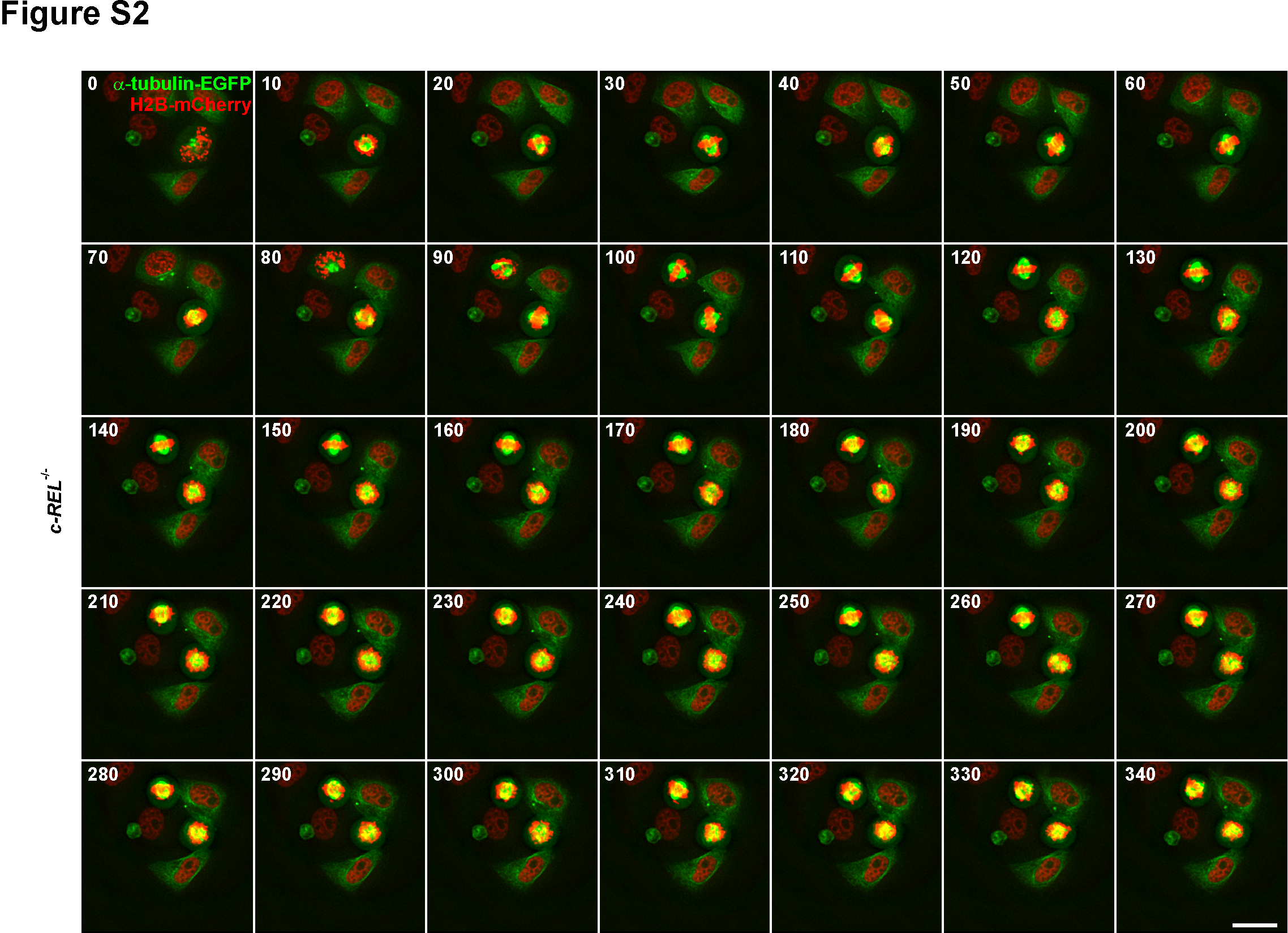

Supplement: S2 Fig — Scale bar: 25 μm. (TIF) [file pone.0182373.s002.tif]

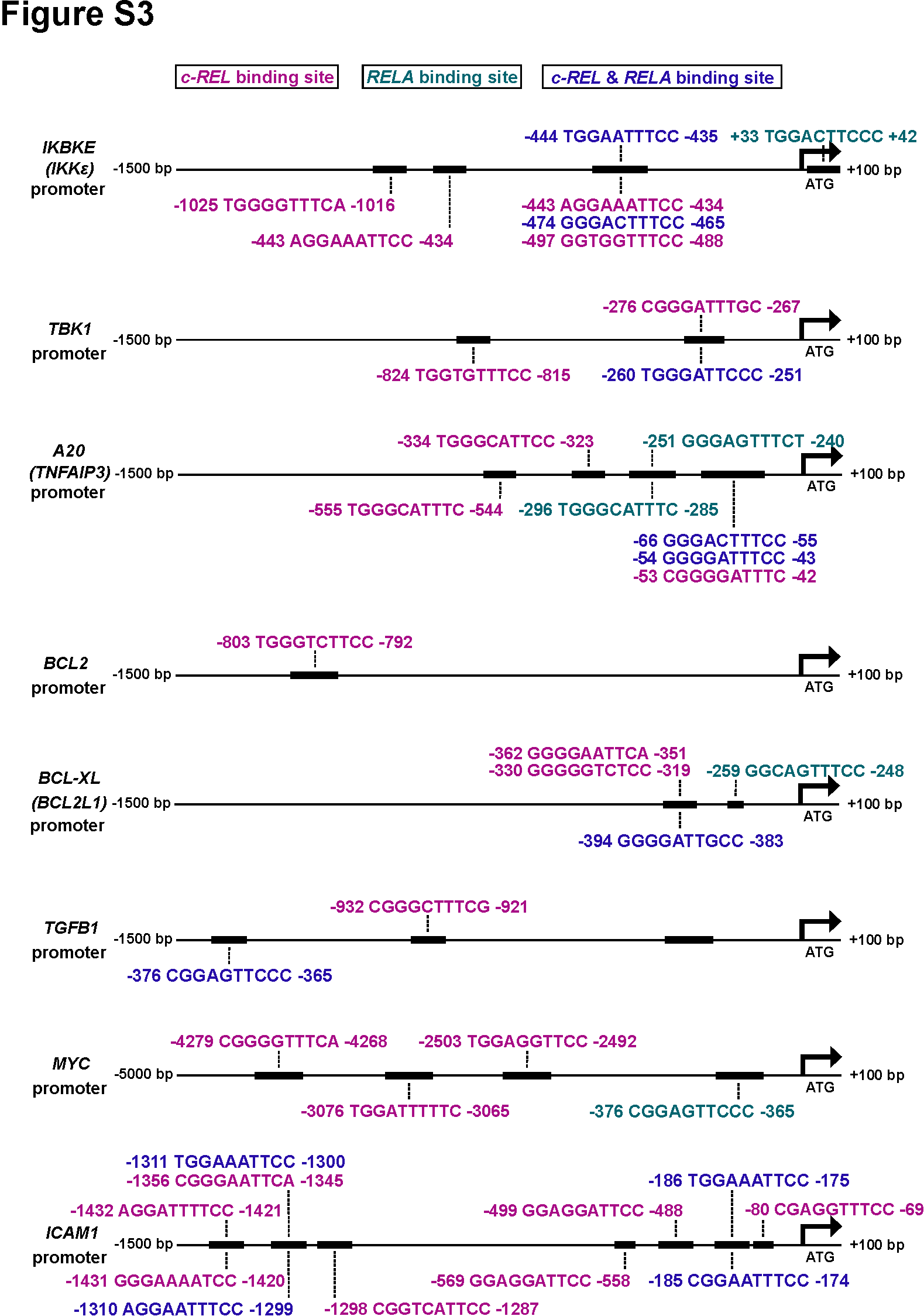

Supplement: S3 Fig — c-REL binding site is shown in magenta, RELA binding site is depicted in cyan and common binding sites are shown in purple. (TIF) [file pone.0182373.s003.tif]
